# Supplementary material for: Venomix: a simple bioinformatic pipeline for identifying and characterizing toxin gene candidates from transcriptomic data
Source: PeerJ. 2018 Jul 31;6:e5361. doi: 10.7717/peerj.5361 (PMC6074769; doi:10.7717/peerj.5361)
Supplement: Supplemental Information 3 [file peerj-06-5361-s003.gz › FinalOutput_E-6/Zinc_metalloproteinase-disintegrin-like_jerdohagin_1/finaltree.pdf]

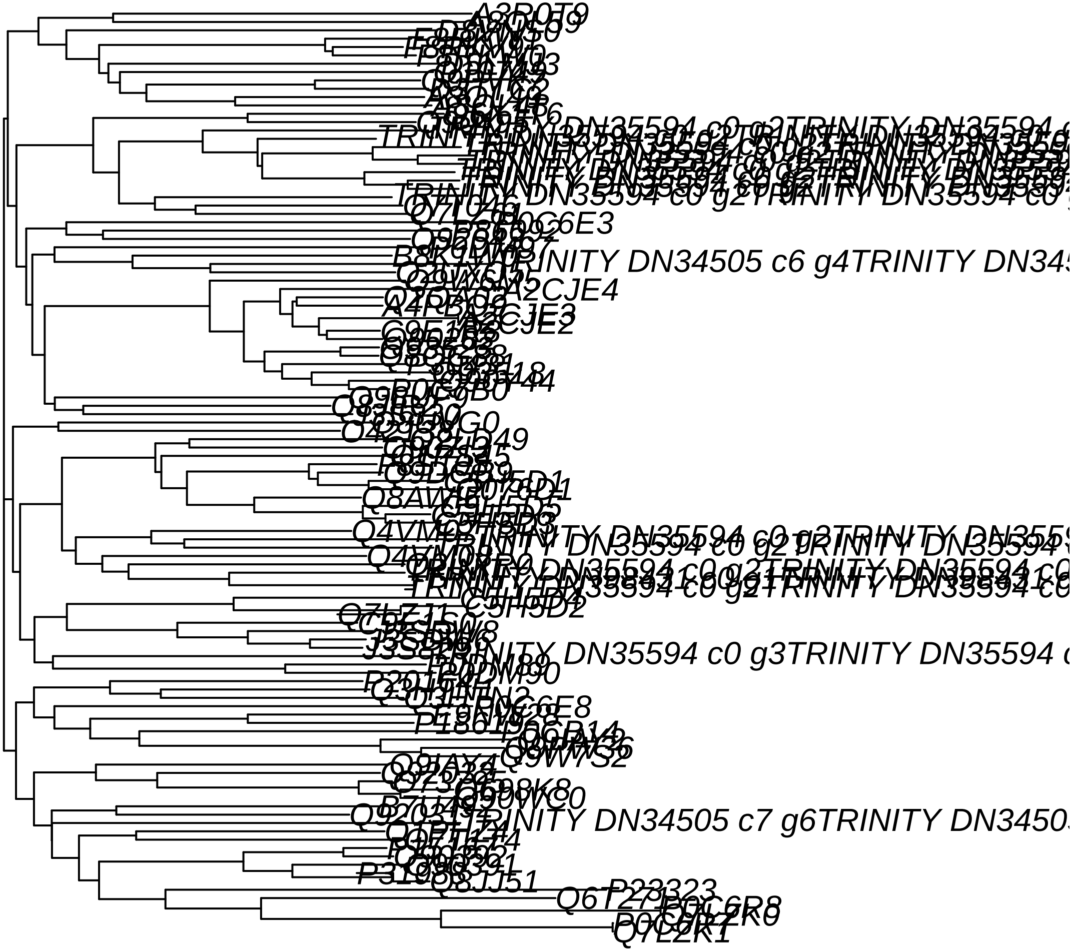
A3R0T9
TRINITY DN34505 c6 g4TRINITY DN345
A2CJE4
A2CJE2
TRINITY DN35594 c0 g3TRINITY DN35594
TRINITY DN34505 c7 g6TRINITY DN3450
C5H5D2
Q8JJ51
P22323
Q7L2K1
